# An exploratory study of GP awareness of carer emotional needs in Western Australia

**DOI:** 10.1186/1471-2296-7-33

**Published:** 2006-05-24

**Authors:** Caroline E Bulsara, Noreen Fynn

**Affiliations:** 1 Primary Health Care Research, Evaluation and Development Unit, General Practice, University of Western Australia, 328 Stirling Highway, Claremont, 6010, Western Australia; 2 Carers WA, 255 Walcott Street, North Perth 6005, Western Australia

## Abstract

**Background:**

The impact of caring for a family member or friend is a life changing experience. Often carers are struggling to cope with ongoing demands of caring for someone. At some point, most carers will approach their family physician for advice on aspects of their role. Carers Western Australia sought information and opinions of General Practitioners (GPs) regarding being a carer. This exploratory survey was to assess the perceptions of Western Australian GPs regarding their role in providing information and support to carers and their awareness of carer needs and issues.

**Methods:**

A telephone survey design of an opportunistic sample of 66 Western Australian GPs was conducted. The responses were both closed and open-ended questions to allow for probing of responses. Responses were analysed using both qualitative and quantitative analysis.

**Results:**

GPs are generally aware of their role in regard to carers and most doctors (88%) said that they had been approached for help in accessing services by carers. A majority of respondents said that carers and spoken to them (70%) and asked for help with (77%) emotional needs. However, when asked how these needs are met, GPs tended to provide practical assistance for the care recipient and carer as a means of addressing those needs. This primarily included providing referral to services to ensure that the carer has practical assistance in caring for the person. However, GPs are less able to provide the necessary emotional or psychological support needed by carers before crisis point is reached. Most doctors said that they had experienced difficulties in providing assistance to carers (84%) at some time.

**Conclusion:**

GPs may be unable or unwilling to provide the necessary assistance to carers who are showing signs of carer burnout and stress. The GP needs to adopt a more holistic approach when treating a patient as to the interaction with the caregiver.

## Background

Research in Australia and overseas has identified the importance to the health system of identifying and supporting unpaid or family caregivers in the community [1-3]. It is further acknowledged that caring for a family member or close friend has an enormous impact upon caregiver health and wellbeing [4,5]. Carers Western Australia [6] are a non government organisation who provide resources, education and support to unpaid or family carers in Western Australia. Carers Western Australia [Carers WA] believe that it was relevant and timely to initiate an exploratory study to gauge the knowledge and awareness of GPs in addressing the needs of caregivers within Western Australia. To date there is very little research in this area from the GPs' perspective. Two smaller studies conducted by Carers WA [6] of its own membership have previously highlighted the importance of the GP as the first point of contact for caregivers when seeking advice and information. As such the role of the GP cannot be overstated [7]. Studies indicate that the GP is a well-placed person to inform and guide caregivers in need of support services [8,9]. Further to this, Carers WA wanted to ascertain the issues and concerns amongst GPs and specialists regarding family or unpaid caregivers. It was hypothesised that although GPs are largely aware of the support services available to caregivers they are less aware of the resources available to address the emotional and psychological needs of caregivers. It is also hypothesised that addressing the psychological stress of caregivers rarely occurs until crisis point has been reached and radical solutions to problems are put into effect. Due to limited funding it was decided to conduct an exploratory study amongst a small number of GPs within Western Australia.

## Methods

An opportunistic sample of 66 GPs and 10 specialist clinicians in Western Australia were obtained from the electronic white pages directory. The use of the telephone white pages is used extensively for cross sectional telephone surveys in Western Australia. GPs (GPs) and specialists who were listed in the Electronic White Pages were telephoned and asked to participate. The researchers endeavoured to obtain a number of rural GPs. Carers WA had requested that ten specialist clinicians be recruited from various fields of specialisation who would be likely to be treating patients with a family/unpaid caregiver. Rural GPs recruited from most major Western Australian regional locations outside the Perth metropolitan area were included the survey. A final sample of 62 metropolitan and 14 rural GPs and specialists were recruited to the study. A telephone survey was conducted with the final sample of GPs and specialists.

In regard to larger practices where more than one GP was listed within the same practice, information was faxed and returned with an expression of interest in participating in the survey at a time convenient for the GP. An appropriate fee remunerating doctors for their time during the telephone interview was offered to those who had agreed to participate.

The questionnaire included both closed questions and open-ended questions to ensure greater depth of information about caregiver and GP/specialist interface. Question areas included GP perceptions of their role in regard to carers, ways in which they provide assistance to carers in terms of referrals and information regarding services and whether carers approach GPs regarding their psychological difficulties in relation to the caring role. GPs were also asked how they provide assistance to carers who are experiencing emotional difficulties in relation to caring and finally whether and at what point GPs would intervene to provide assistance to carers who are experiencing emotional and psychological stress. Questions to ascertain demographics of respondents were also included in the questionnaire. The results were analysed using SPSS Version 11.0 and QSR NVivo for coding and categorising open-ended responses into themes. Open ended response were recorded verbatim and are used as quotes to highlight pertinent issues. The results of this study will focus solely on the findings and views in regard to the sample of 66 GPs rather than including the views of the small sample of specialists. Furthermore, the issues and concerns of specialists are very different from those of the GP and should be dealt with in a separate forum. Both quantitative and qualitative findings will be presented in this article.

## Results

Although GPs were able to define their role in relation to assisting the carer, many defined this as the provision of more services for the care recipient and less often to referring caregivers on to psychological support services and community support services. In other words, addressing carer needs was equated to providing further services to enable to caregiver to fulfil their caring role. GPs although aware of caregiver emotional needs, were less able to effectively refer the carer on to support services.

### GP demographics

Overall, most GPs in the study (80%) practised in the Perth Metropolitan region. A majority of GPs (60%) had spent at least 16 years as a GP and had been in their current location (practice setting) for less than ten years (60%). GPs had interests in the areas of mental health (13%), geriatric medicine (20%), paediatrics (10%) and women's health (13%).

### GP perceptions of caregiver demographics

Of the total GP respondents, 75% believed that only 20% of their total number of patients had a family carer.

GPs said that they were frequently asked to refer caregivers onto services but this usually meant provision of information or referral onto Home Nursing Services (38%) or an Aged Care Assessment Team (ACAT) (27%). Psychiatric service referrals for carers accounted for 5% of total referrals.

### GP ability to assist caregivers with their own needs

Although, 96% of GPs believed that they were able to assist with caregiver needs, Table 1 demonstrates that this is largely in terms of information (89%) and referrals to services (86%) for the care recipient.

**Table 1:** Types of assistance provided by GPs

Response (*n = 66*)	***n***	% of respondents (95% CI)*
Information on services	59	89% (79–96%)
Referral to services (support groups, community services)	57	86% (76–94%)
Respite	57	86% (76–94%)
Medication	55	83% (72–91%)
Counselling	51	77% (65–87%)
Family support	45	68% (56–79%)
Referral to specialists	43	65% (52–76%)
Other	16	24% (15–36%)

*Exact confidence intervals for samples from Binomial Distribution

In terms of psychological support for the carer, 77% said that they would provide counselling for carers. However, when asked to describe what they meant by 'counselling' this was described as 'lending a listening ear' by two GPs and 45% said that they would counsel the carer themselves. This is a concern because some carers may be in need of formal counselling sessions and are not being referred on to those services. In fact, further to this, only one GP in our sample had a formal counselling qualification.

Overall, 57% of GPs said that caregivers tended to talk about their own needs solely in relation to the care recipient. The remaining 42% said that they would tend to talk exclusively about the needs of the care recipient. Notwithstanding, 70% of family doctors said that they were asked for help with caregivers' emotional needs by caregivers themselves at some point. In response to this, GPs were asked if they would ever intervene when it became clear that the caregiver was experiencing extreme difficulty in coping. Two doctors highlighted the difficulties with accessing counselling services outside of the private sector as the main problem. Only 16% of GPs referred patients to community health centre counsellors and 14% to a clinical psychologist (private patients only).

### Provision of emotional support

GPs preferred to assist with emotional needs by offering practical ways of helping with the duties of the caregiver. These needs were met by referring carers to community services Overall, twenty one doctors noted respite as a frequently offered means of helping caregivers cope with emotional burnout and ranged from a brief 'going for a walk' to a lengthier break when 'going on holiday'. Respite in terms of protecting the caregiver's health was also highlighted.

GPs noted the difficulties in addressing caregiver needs due to the fact that caregivers generally talk more about the care recipient than themselves. For this reason, doctors become adept at reading body language signals from caregivers as to when they are not coping well. For some doctors this makes it difficult to pinpoint caregiver problems. As one GP put it,

I take my cues from how they look and their body language tells me they need to be asked how they are feeling.

One GP believed that caregiver difficulties were often "couched in physical need" and had to be 'looked out for'. In fact only 9% of GPs said that caregivers had asked for a referral to psychological services. This was particularly true of psychological problems such as depression. Depression is also common amongst caregivers known to the GP. Assistance in dealing with depression usually involves referral to a counsellor or social worker and medication such as anti depressants.

The implication is that they are looking for medication help by not specifically saying that but it is implied. That is what they need referring to stress related problems.

Sometimes they just say they are depressed. It is a cry for help. They see no way of resolving the situation without being in a moral or ethical dilemma.

In relation to the GP's ability to respond to emotional needs of caregivers, some saw also their role as counsellor and mediator in conflict resolution between family members and care recipient. Some GPs said that for caregivers 'just letting a bit out" was enough in terms of counselling and for others it was a combination of GP counselling, psychologist and support group.

Counselling, support groups and sometimes medication and to talk to someone, who knows who has been through what they are going through.

One GP took this role further in advocating on behalf of the caregiver and reinforcing the value of their role. This included reassuring the caregiver that they are not alone and are "doing a good job". Three further GPs noted the need for recognition and acknowledging the caregiver for the role amongst the wider community.

Many caregivers were believed by GPs to struggle with the ongoing emotional demands of the caregiving role. Furthermore, a sense of guilt that many experience when they ask for assistance or admit that they are having difficulty in coping add to the emotional demands of the role. One GP highlighted the importance of providing the caregiver with the ability to set limits and establish boundaries in terms of time and input. This entails encouraging the caregiver to move beyond the feelings of guilt and acknowledging feelings such as resentment that the person whom the caregiver is caring for is no longer the same or parents feeling guilty and that the caregiver is in some way responsible when a child has a conduct disorder or drug problem. Part of the guilt for many caregivers was perceived by doctors to be a result of feelings of frustration over the caregiving role. One GP explained,

It impacts on family relationships and how a son or daughter relate to their partner. It impacts if behavioural changes in the patient are there in terms of partner not leaving the house and going to the day care centre. The care recipient wants them there all the time.

## Discussion

Cohen, C.A [7] noted that there was a role for family physicians in following caregivers longitudinally to ensure support and wellbeing are maintained. Although our exploratory survey revealed that most doctors were aware of services for caregivers, the support provided was of a completely practical nature in terms of caring for the patient. When psychological support for the caregiver was directly offered by the GP it was of an informal sharing or 'coffee cup counselling' nature. Caregiver stress and the effects of social isolation [10,11] are a common and often neglected area of mental health. A study by Burns et al [12] indicated that brief primary care interventions were successful in alleviating caregiver stress. The study further indicated that dealing with care recipient behaviour without addressing caregiver issues, may not be enough to ensure that caregiver stress is lessened.

When caregivers asked for emotional help GPs usually prescribed medication for depression related problems and in severe cases a referral to a psychiatrist. Caregiver stress is further exacerbated by problems regarding limitations of shorter consultation time in current general practice. Mental health problems are ill suited to resolution within the time bound primary health care setting. An intervention study by Mazonson et al [13] requested the patient to complete a self-reported anxiety questionnaire whilst in the waiting room. Results revealed that clinicians had increased recognition when receiving feedback on anxiety levels amongst patients and were more likely to refer previously unrecognised patients experiencing anxiety. There may be a potential in this area for doctors or possibly practice nurses to identify carers experiencing stress to be referred on to support groups or services through the GP which assist in alleviating anxiety.

### GP awareness of their role in relation to the caregiver

It was evident that some GPs saw their role as a purely practical one in terms of referring and directing caregivers to appropriate services. Others regarded their role equally as a supportive one. In particular, rural GPs viewed their role as supportive and were more likely to show concern for caregivers in terms of ability to maintain contact and interaction with the rest of the community. In other words, caregiver concerns became community concerns. All GPs noted the importance of being able to provide practical support for caregivers. Respite was the key concern and GPs strove to ensure that caregivers were 'getting enough breaks' from their caring role.

Furthermore, doctors spoke of having to 'coax the caregiver' at times to take respite breaks. Support needs for caregivers were divided into two categories. Support was viewed on a financial level and also on an emotional level by many doctors. Part of ensuring financial support involved determining any difficulties or needs which had arisen for a caregiver and their family. In terms of financial support, making certain that caregivers received all entitlements and caregiver payments was perceived by most respondents as extremely important.

Another issue which has been further highlighted in this study is the sense that community services and support groups are largely underutilised within the primary health care system. Most GPs also highlighted the fact that they had experienced difficulties in accessing services for caregivers at some time. This combined with the time factor for the standard ten minute GP consultation culminate in a need to deal with problems arising expediently.

Difficulties also existed when GPs felt unable to intervene to assist a caregiver who was not coping well with the demands of the caring role until crisis point had been reached. This may be due to the fact that carers are reluctant to accept help when concerned that they may be perceived as 'not coping'. Thus the GP is unable to intervene until it was evident to the GP that the carer was undergoing severe stress or the general health of the care recipient was deteriorating.

## Limitations

This was an exploratory study of GPs and specialists in Western Australia. As such the numbers are limited, particularly in the case of the specialists. However, the GPs were opportunistically selected using the electronic white pages and the Computer Assisted Interview system and invited to participate.

## Conclusion

The study highlights the need for a more caregiver focussed model to deal with the stress and anxiety associated with the caregiving role. Our study revealed that for most doctors, however, it is currently a case of dealing with problems and crises ad hoc rather than planning in advance. Given that the GP is the first point of contact for many caregivers this may be an issue which requires further investigation in assisting GPs to better cope with caregiver mental health needs. In relation to this, it is suggested that longer consultation times are often needed for the carer and care recipient. Furthermore, a review of the health system mechanism is required to ensure that carers have the community support services, psychological support services that they need and a reduction in the unnecessary prescribing of medications for anxiety and depression which may be preventable. It also requires better GP education in terms of the causes and signs of carer burnout and how to provide education to carers in how to cope with their caregiving role and ensure that they are also take care of themselves.

## Abbreviations

GP – General Practitioner

Carers WA – Carers Western Australia.

## Competing interests

The author(s) declare that they have no competing interests.

## Authors' contributions

Noreen Fynn and Caroline Bulsara – Conceiving and designing the study, obtaining funding, and revising the report.

Caroline Bulsara – Collecting and analysing data, interpreting the data and writing the report.

## Pre-publication history

The pre-publication history for this paper can be accessed here:


